# Comparison of Streptococcus mutans Adhesion on New and Recycled Metal Brackets: An In Vitro Study

**DOI:** 10.7759/cureus.23574

**Published:** 2022-03-28

**Authors:** Vasumathi Thiruvengadam, Arun B Chitharanjan, Kennedy Kumar, Venkatesan Singaram

**Affiliations:** 1 Orthodontics, Panimalar Medical College Hospital & Research Institute, Chennai, IND; 2 Orthodontics, Retired, Chennai, IND; 3 Microbiology, Sri Ramachandra Institute of Higher Education and Research, Chennai, IND; 4 Sri Ramachandra Faculty of Engineering and Technology, Sri Ramachandra Institute of Higher Education and Research, Chennai, IND

**Keywords:** flame heating, ultrasonic cleaning, acid bath, sandblast and electropolish, streptococcus mutans adhesion

## Abstract

Background: Evaluation of adhesion of *Streptococcus mutans* on recycled orthodontic brackets is significant, as *Streptococcus mutans *is the main causative factor in enamel demineralization and many clinicians, in their practice, resort to reconditioning of brackets, as it is cost-effective. Thus, this study aimed to evaluate and compare the adhesion of *Streptococcus mutans* on new brackets of three different companies (Group I, Group II, and Group III) and brackets recycled by three different recycling methods (RC I: flame heating followed by acid bath; RC II: flame heating followed by ultrasonic cleaning and electropolishing; RC III: flame heating followed by sandblasting and electropolishing).

Materials and methods: A total of 10 brackets from each group were incubated with 10^8^ colony-forming units (CFUs) of *Streptococcus mutans* in trypticase soy broth overnight. The brackets were then washed with phosphate-buffered saline and treated with 0.25% trypsin for 20 minutes followed by vertexing the solution to remove the adhered bacteria and then the solution was plated on the blood agar and incubated overnight. The total viable count of bacteria was quantified.

Results: Comparing all the three groups and recycling methods, Group II brackets showed significantly more adhesion, Group I brackets showed lesser adhesion, and Group III brackets showed intermediate adhesion. When comparing recycling methods, all the three methods of recycling with all the three groups showed more bacterial adhesion than the control brackets, which was statistically insignificant (P > 0.05).

Conclusion: Recycled brackets showed more bacterial adhesion and electropolishing resulted in reduced bacterial adhesion.

## Introduction

The crux of orthodontic practice involves fixed appliance therapy, which controls the tooth in three-dimensional axes, to achieve proper occlusion and root parallelism. To meet this requirement, bonded brackets should function throughout the course of treatment. Unfortunately, bond failure may manifest before the completion of treatment, necessitating rebonding in the midst of treatment to achieve the ideal tooth position. Besides bond failure, bracket repositioning during treatment could also necessitate the need for rebonding. Recycling of brackets removes adhesive remnants from the bracket base without distortion of bracket surfaces and tie wings, thereby permitting the same bracket to function as a new bracket without compromising much on the bond strength.

Although several commercial recycling methods are available [1-5], the chairside execution of such methods is impractical [5]. This led to the design of many in-office recycling methods such as low-speed air rotor grinding of the adhesives using greenstone [4,6,7] and silicon carbide bur [8], air abrasion using aluminum oxide particles [6-8], direct flaming followed by sandblasting and electropolishing [3], direct flaming followed by ultrasonic cleaning [7] and electropolishing [9], and direct flaming followed by immersion in the acid bath [10].

The complexity of orthodontic brackets and their long periods of attachment to the teeth provide an environment for plaque formation and bacterial adhesion, principally *Streptococcus mutans* with subsequent enamel demineralization [11,12]. Orthodontic adhesives showed more bacterial adhesion than brackets because of increased surface free energy [13,14]. This difference in bacterial adhesion is attributed more to the composition of the bracket rather than its type [15]. Adhesion of *Streptococcus mutans* was more in plastic brackets when compared to metal brackets [16,17]. This is contrary to another study that showed a statistically insignificant comparison [18-20]. Few studies showed that saliva coating reduces bacterial adhesion [17,21].

Previous studies on bracket recycling focused primarily on bond strength and clinical survival rate [1,4-8], corrosion resistance [2,3], ion release [22,23], and slot tolerance [6]. Brackets that form an integral part of fixed appliance therapy increase bacterial adhesion. To our knowledge, the influence of recycling methods on bacterial adhesion has not been dealt with in any of the previous studies. Thus, the aim of this study is to evaluate and compare *Streptococcus mutans* adhesion on new metal brackets with three different methods of recycled brackets.

## Materials and methods

An in vitro study was conducted in a tertiary care center after procuring institutional ethics approval from the Department of Orthodontics & Dentofacial Orthopedics and Microbiology (reference number: CSP/13/JUN/29/123). The sample consisted of a total of 120 lower right second premolar brackets from three different companies, each comprising 40 brackets; these were comprised into four broad groups, as shown in Table 1.

**Table 1:** Sample groups

Groups	Control: New brackets	RC I: Flame heating and acid bath	RC II: Ultrasonic cleaning with electropolishing	RC III: Sandblasting with electropolishing
Group I	10	10	10	10
Group II	10	10	10	10
Group III	10	10	10	10

Inclusion and exclusion criteria and segregation of sample

A total of 90 human lower premolars, therapeutically extracted for orthodontic treatment, were collected from the Department of Oral and Maxillofacial Surgery, Faculty of Dental Sciences, Sri Ramachandra University, and from a few private clinics. The tooth selection included an inclusion criterion, which was the age group between 15 and 22 years comprising both males and females. The exclusion criteria were teeth having signs of attrition and abrasion, teeth having caries lesions, and teeth having any visible cracks.

As per the American Dental Association guidelines on handling extracted teeth, the visible blood and gross debris were cleaned from the teeth and immersed in a 10% formalin solution for seven days, which was effective in disinfecting both internal and external structures of the teeth. Then the teeth were stored in saline solution (0.9% NaCl) until used for study purposes. All the 90 teeth were mounted on plaster blocks of 10 teeth each in such a way that the crowns of the teeth were exposed for bonding. Thus, nine separate blocks were randomly segregated into nine sub-groups, namely, Group I-RC I, Group I-RC II, Group I-RC III, Group II-RC I, Group II-RC II, Group II- RC III, Group III-RC I, Group III-RC II, and Group III-RC III, based on the bracket company and recycling method used in this study, as shown in Table 1.

Study design

All the brackets were bonded using the following procedure. The buccal surface of the teeth crowns was polished with pumice and a rubber cup, followed by etching for 15 seconds with 37% phosphoric acid gel (Prime Dental Products Pvt Ltd, Thane, India), then washed for 30 seconds and air-dried. Next, the bonding agent was applied (Ortho Solo Universal Bond Enhancer, SDS Ormco, Ormco Corporation, Glendora, CA) and cured for 20 seconds with a halogen light cure system, with the intensity of 480 nm (visible light cure unit, 3M ESPE, 3M, Saint Paul, MN). Brackets were then bonded using Enlight Light Cure Adhesive (Ormco Corporation) and cured for 40 seconds with the same halogen light cure system, with an intensity of 480 nm (visible light cure unit, 3M ESPE). Brackets were debonded by shear force applied with the blades of the posterior debonding plier in groups for the recycling procedures.

**Figure 1:**
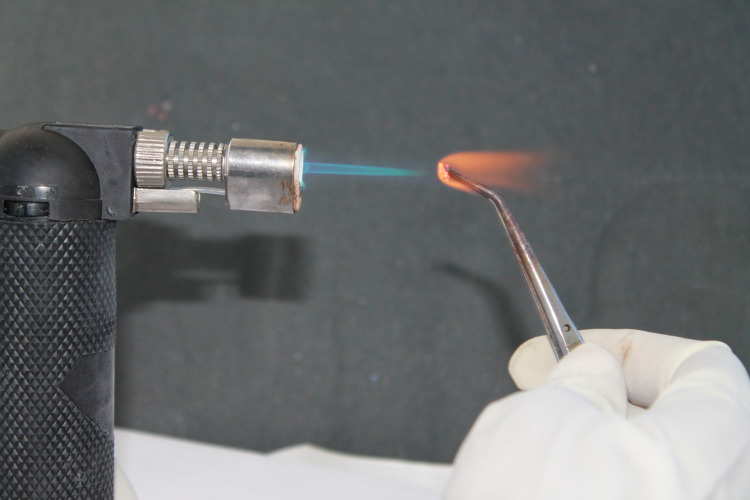
Flame heating

**Figure 2:**
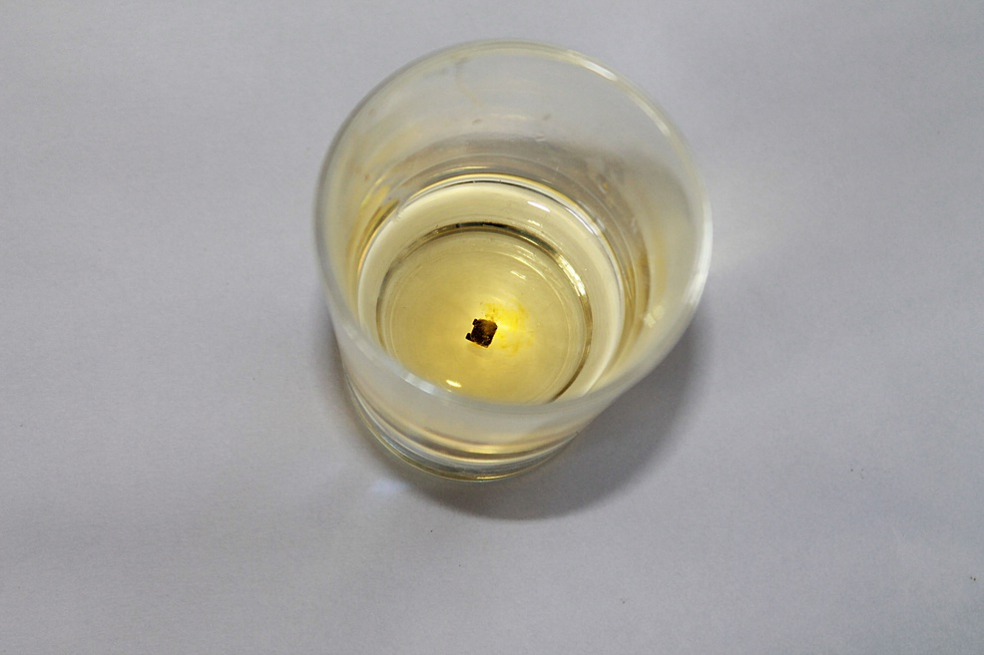
Acid bath

Brackets were recycled with three different methods. The first method was flame heating (Figure 1), followed by an acid bath (Figure 2) (recycling method I, RC I). The adhesive was burned off for 15-20 seconds from each bracket using a soldering torch and each bracket was submerged for 10-15 seconds in a solution of 32% hydrochloric acid and 55% nitric acid mixed in a 1:4 ratio and washed in running water for 30-40 seconds.

**Figure 3:**
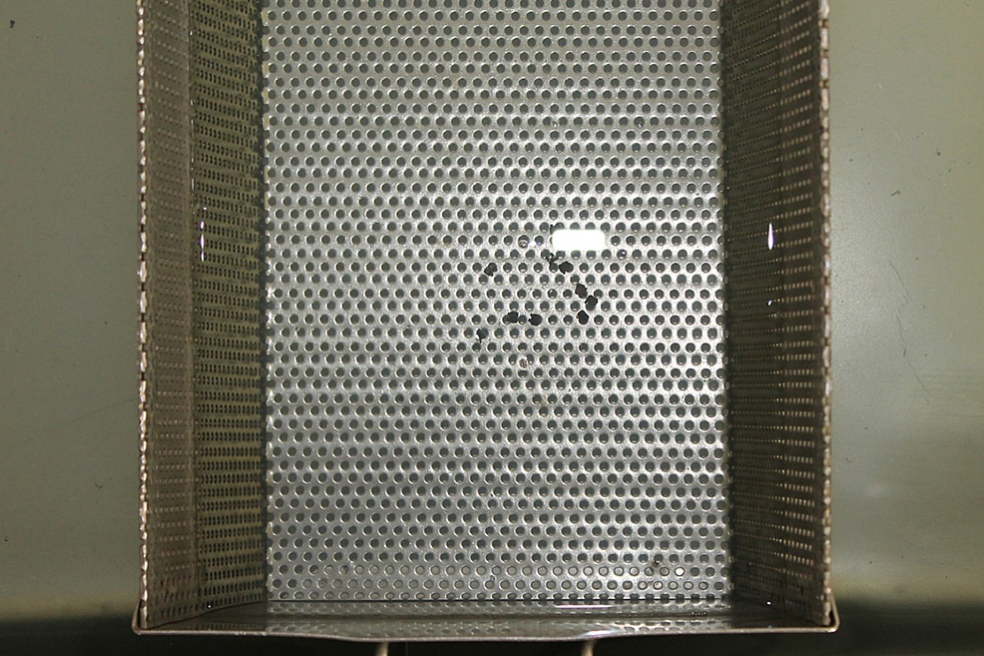
Ultrasonic cleaning

**Figure 4:**
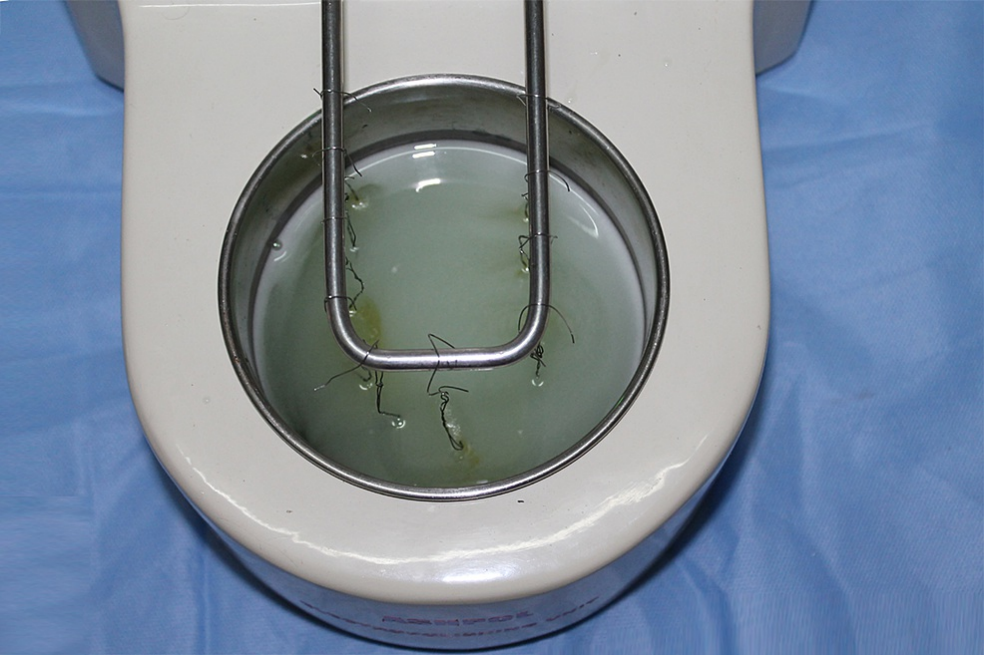
Electropolishing

The second method was ultrasonic cleaning (Figure 3) with electropolishing (Figure 4) (recycling method II, RC II). The adhesive was burned off from each bracket for 15-20 seconds using a soldering torch, and then the entire group of 10 brackets was ultrasonically cleaned for 10 minutes (Confident Ultrasonic Cleaner, Confident, Bengaluru, India) to remove the adhesive remnant and air-dried, and then the entire group of 10 brackets was electropolished by hanging the brackets from the upper U electrode rod using 24 gauge soft stainless steel wire into an electrolytic solution at 5 amps and 220V power for two minutes (Ashpol Electropolishing Unit, Jaypee General Agencies, Calicut, India).

**Figure 5:**
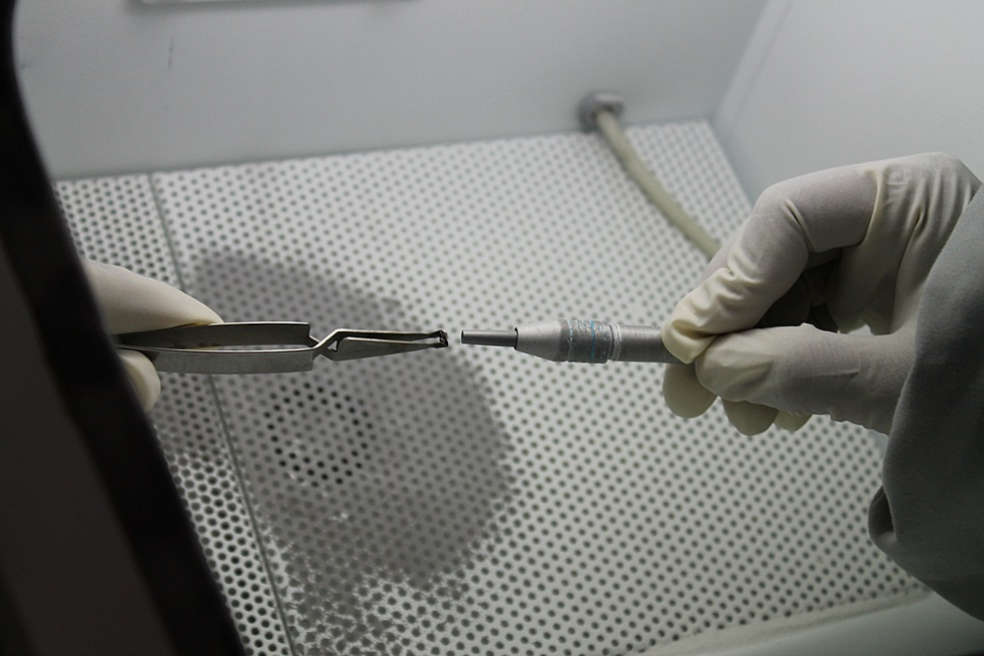
Sandblasting

The third method was sandblasting (Figure 5) with electropolishing (recycling method III, RC III). The adhesive was burned off for three to five seconds from each bracket until the bonding agent started to ignite and burn, followed by sandblasting for 10-20 seconds using 90 µm aluminum oxide particles with 90 PSI pressure. The brackets were held at approximately 10 mm from the tip of the etcher at 900 angulations (Ideal Blaster Sandblasting Unit, Delta Labs, Chennai, India) and the entire group of 10 brackets was electropolished for two minutes.

Assessment of bacterial adhesion

**Figure 6:**
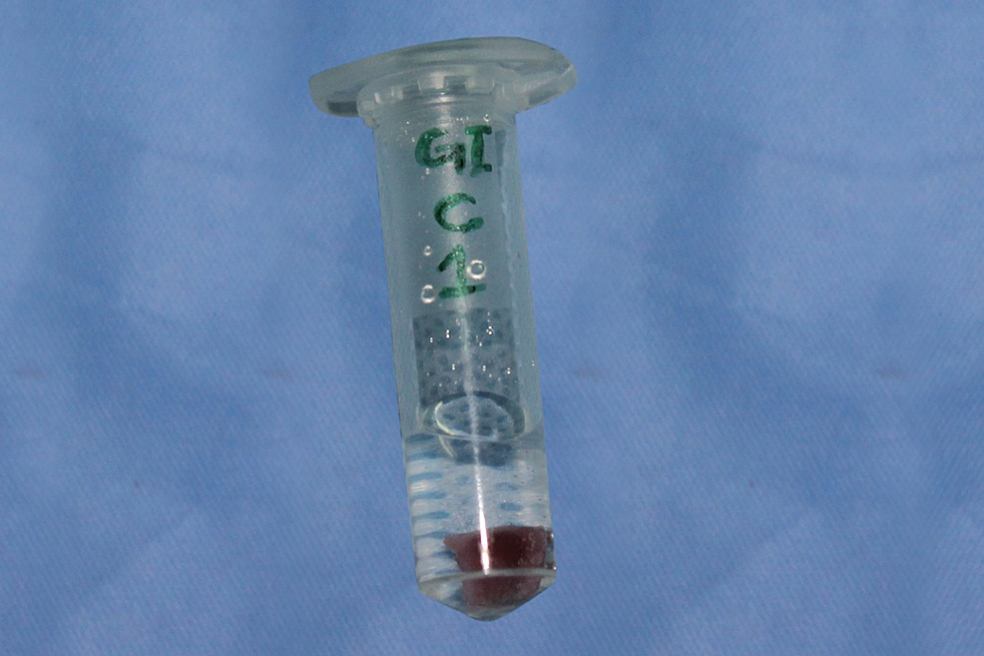
Sample

In vitro assessment of *Streptococcus mutans *adhesion on new and recycled brackets was done in the Department of Microbiology. The bracket base was covered with red utility wax to avoid the adhesion of bacteria on the base surface. The brackets were sterilized by the ethylene oxide sterilization method in Central Sterile Supply Department (CSSD). Pure strains of *Streptococcus mutans* (Microbial Type Culture Collection and Gene Bank (MTCC): 497) were commercially procured from MTCC, Chandigarh in the form of freeze-dried powder and reconstituted as per the instructions given by the manufacturers. *Streptococcus mutans* suspension of 10^8^ colony-forming units (CFUs) was prepared using McFarland standards; McFarland equivalence standards are intended to be part of a quality control program for adjusting densities of bacterial suspensions that are used for identification and susceptibility testing. Each bracket was first taken in a 2 ml Eppendorf tube having 1 ml trypticase soy broth to which 10 microliters of *Streptococcus mutans* suspension (10^8 ^CFUs) was added (Figure 6). The same was incubated at 37°C in 5% CO_2 _for 24 hours, with intermittent shaking. After incubation, the bracket was aseptically taken out of the tube using sterile forceps and washed with sterile phosphate-buffered saline (PBS) solution to remove excess nonadherent bacteria after which the wax sheet from the bracket base was removed. Then the bracket was treated with 0.5 ml of 0.25% trypsin for 20 minutes followed by vertexing for two minutes at 2000 rpm to remove the adherent bacteria (REMI Cyclo Mixer, REMI, Mumbai, India). The trypsin solution obtained after vertexing was plated directly into 5% sheep blood agar and diluted to 10^-1 ^fold (0.1 ml of vertexed trypsin solution with 0.9 ml brain heart infusion (BHI) broth) and then plated into 5% sheep blood agar plate to see the viable adherent bacteria. The plates were incubated overnight at 37°C in a 5% CO_2_ incubator.

Data collection

**Figure 7:**
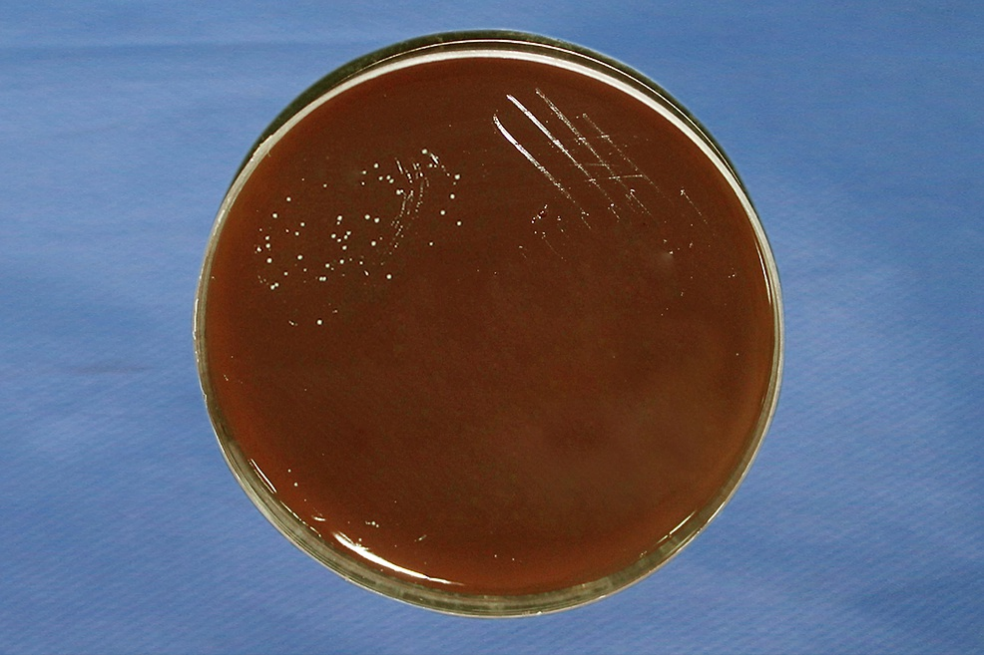
Bacterial colonies

Reading was taken after 24 hours of incubation, wherein growth in 5% sheep blood agar was counted by visual colony counting (Figure 7). Gram stain was done to confirm the growth of *Streptococcus mutans*. In each group, 10 brackets were tested and the mean colony count was taken for statistical analysis.

## Results

Statistical analysis

Mean and standard deviations were calculated from the readings and the number of CFUs using SPSS software (IBM Corp., Armonk, NY). To find the significant difference in the multivariate analysis, analysis of variance (ANOVA) with Tukey's honest significant difference (HSD) post hoc test was used.

Determination of bacterial adhesion

The descriptive data showed the mean S*treptococcus mutans* adhesion (CFUs), as shown in Table 2.

**Table 2:** Comparison of the mean Streptococcus mutans (in colony-forming units) of the various groups

		N	Mean	Std. deviation	Std. error	95% confidence interval for mean		Minimum	Maximum	
						Lower bound	Upper bound			
Controls	Group I	10	79.250	5.8417	1.8473	75.071	83.429	66.5	85.0	
	Group II	10	108.450	12.3141	3.8940	99.641	117.259	91.5	128.0	
	Group III	10	90.700	4.9453	1.5638	87.162	94.238	83.0	98.5	
	Total	10	92.800	14.6468	2.6741	87.331	98.269	66.5	128.0	
Group I	Group I	10	85.700	4.5656	1.4438	82.434	88.966	79.0	95.0	
	Group II	10	113.500	9.1803	2.9031	106.933	120.067	101.5	128.0	
	Group III	10	94.300	4.1245	1.3043	91.350	97.250	87.5	101.0	
	Total	10	97.833	13.3270	2.4332	92.857	102.810	79.0	128.0	
Group II	Group I	10	83.850	7.1104	2.2485	78.763	88.937	73.5	97.5	
	Group II	10	110.200	7.7035	2.4361	104.689	115.711	101.0	128.0	
	Group III	10	93.950	5.5400	1.7519	89.987	97.913	86.5	102.5	
	Total	10	96.000	12.8654	2.3489	91.196	100.804	73.5	128.0	
Group III	Group I	10	84.300	7.4692	2.3620	78.957	89.643	74.0	96.0	
	Group II	10	110.200	8.7882	2.7791	103.913	116.487	101.0	128.0	
	Group III	10	93.300	5.7648	1.8230	89.176	97.424	86.0	103.0	
	Total	10	95.933	13.0706	2.3864	91.053	100.814	74.0	128.0	

Comparison of bacterial adhesion

Comparing the *Streptococcus mutans* adhesion, the results of the ANOVA indicated a significant difference between groups (P < 0.05) (Table 3).

**Table 3:** One-way analysis of variance (ANOVA)

		Sum of squares	df	Mean square	F	Sig.
Controls	Between groups	4329.350	2	2164.675	30.892	0.000
	Within groups	1891.950	27	70.072		
	Total	6221.300	29			
RC I	Between groups	4051.467	2	2025.733	49.759	0.000
	Within groups	1099.200	27	40.711		
	Total	5150.667	29			
RC II	Between groups	3534.650	2	1767.325	37.711	0.000
	Within groups	1265.350	27	46.865		
	Total	4800.000	29			
RC III	Between groups	3458.067	2	1729.033	31.200	0.000
	Within groups	1496.300	27	55.419		
	Total	4954.367	29			

Multiple comparisons between the groups with the post hoc Tukey HSD test (Table 4) revealed that Group I control showed significantly lesser adhesion when compared to Group II (P = 0.000) and Group III controls (P = 0.013), Group III control showed more adhesion than Group I control and lesser adhesion than Group II control (P = 0.000), and Group II control showed higher adhesion than Group I and Group III controls.

**Table 4:** Multiple comparisons between the groups with post hoc Tukey honest significant difference test

Dependent variable	(I) Groups	(J) Groups	Mean difference (I-J)	Std. error	Sig	95% confidence interval	
						Lower bound	Upper bound
Controls	Group I	Group II	−29.2000	3.7436	0.000	−38.482	−19.918
		Group III	−11.4500	3.7436	0.013	−20.732	−2.168
	Group II	Group I	29.2000	3.7436	0.000	19.918	38.482
		Group III	17.7500	3.7436	0.000	8.468	27.032
	Group III	Group I	11.4500	3.7436	0.013	2.168	20.732
		Group II	−17.7500	3.7436	0.000	−27.032	−8.468
RC I	Group I	Group II	−27.8000	2.8535	0.000	−34.875	−20.725
		Group III	−8.6000	2.8535	0.015	−15.675	−1.525
	Group II	Group I	27.8000	2.8535	0.000	20.725	34.875
		Group III	19.2000	2.8535	0.000	12.125	26.275
	Group III	Group I	8.6000	2.8535	0.015	1.525	15.675
		Group II	−19.2000	2.8535	0.000	−26.275	−12.125
RC II	Group I	Group II	−26.3500	3.0615	0.000	−33.941	−18.759
		Group III	−10.1000	3.0615	0.007	−17.691	−2.509
	Group II	Group I	26.3500	3.0615	0.000	18.759	33.941
		Group III	16.2500	3.0615	0.000	8.659	23.841
	Group III	Group I	10.1000	3.0615	0.007	2.509	17.691
		Group II	−16.2500	3.0615	0.000	−23.841	−8.659
RC III	Group I	Group II	−25.9000	3.3292	0.000	−34.155	−17.645
		Group III	−9.0000	3.3292	0.031	−17.255	−745
	Group II	Group I	25.9000	3.3292	0.000	17.645	34.155
		Group III	16.9000	3.3292	0.000	8.645	25.155
	Group III	Group I	9.0000	3.3292	0.031	0.745	17.255
		Group II	−16.9000	3.3292	0.000	−25.155	−8.645

When comparing recycling method I (RC I: flame heating and acid bath) among the groups, Group I-RC I showed significantly lesser adhesion than Group II-RC I (P = 0.000) and Group III-RC I (P = 0.015), Group III-RC I showed significantly lesser adhesion than Group II-RC I (P = 0.000) whereas more adhesion than Group I-RC I, and Group II-RC I showed significantly more adhesion than Group I-RC I and Group III-RC I.

When comparing recycling method II (RC II: ultrasonic cleaning with electropolishing) among the groups, Group I-RC II showed significantly lesser adhesion than Group II-RC II (P = 0.000 ) and Group III-RC II ( P = 0.007), Group III- RC II showed higher adhesion than Group I-RC II and lesser adhesion than Group II-RC II (P = 0.000), whereas Group II-RC II showed significantly more adhesion than Group I-RC II and Group III-RC II.

When comparing recycling method III (RC III: sandblasting and electropolishing) among the groups, Group I-RC III showed significantly lesser adhesion than Group II-RC III (P = 0.000) and Group III-RC III (P = 0.031), Group III-RC III showed higher adhesion than Group I-RC III and significantly lesser adhesion than Group II-RC III (P = 0.000), whereas Group II-RC III showed significantly higher adhesion than Group I-RC III and Group III-RC III.

In the overall comparison between groups with new and recycling methods, Group II showed higher adhesion, Group I showed lesser adhesion, whereas Group III showed intermediate adhesion between Group I and Group II.

When comparing the *Streptococcus mutans *adhesion within the groups, results of the ANOVA indicated there was no statistically significant difference seen (P > 0.05) (Table 5).

**Table 5:** One-way analysis of variance (ANOVA) within groups (control and recycling methods)

			Sum of squares	df	Mean square	F	Sig.
	Group I	Between groups	234.625	3	78.208	1.939	0.141
		Within groups	1451.850	36	40.329		
		Total	1686.475	39			
	Group II	Between groups	133.519	3	44.506	0.478	0.700
		Within groups	3352.425	36	93.123		
		Total	3485.944	39			
	Group III	Between groups	79.569	3	26.523	1.007	0.401
		Within groups	948.525	36	26.348		
		Total	1028.094	39			

In all the groups, the comparison of *Streptococcus mutan*s adhesion within the groups (control and recycling methods) was not statistically significant, which revealed that the recycling methods used in the study had not significantly increased the *Streptococcus mutans *adhesion.

## Discussion

Previous studies showed that when compared to other teeth, lower second premolars showed more failure rate [23,24] because of possible moisture contamination during bonding [23,24], higher masticatory forces [23-25], larger aprismatic enamel in the premolars [23], and partially erupted second premolars at the time of bonding [1]. Hence, the lower right second premolar brackets were used in the present study.

Few of the previous studies mimicked recycled brackets by applying the adhesive at the bracket base followed by heating and burning off the same [22,23]. But this method did not ensure exact simulation of a clinical situation. To accomplish this, a cycle of bonding and debonding was performed. Dawjee and Gheevargheses [10] described the recycling method I (RC I), which was a direct flaming and acid bath technique, as a simple and quick method that effectively removed the tarnish caused by heating, thus eliminating the need for an electropolishing procedure.

However, the acid used was 32% hydrochloric acid and 55% nitric acid mixed in a 1:4 ratio. Hence, it should be handled with care [10]. According to the present study, the acid bath method of recycling showed more *Streptococcus mutans* adhesion in all groups of brackets than the other two methods, which involved electropolishing. This may be due to the change in the surface characteristics of the bracket after the acid bath, which requires further investigation.

In the flame heating followed by ultrasonic cleaning and electropolishing method (RC II), an earlier study by Quick et al. [7] suggested that flame heating for 10 seconds and ultrasonic cleaning for five minutes was insufficient to dislodge the residue. Hence, in our present study, the adhesives were burnt off completely for 15-20 seconds and ultrasonically cleaned for 10 minutes to dislodge the residues, followed by electropolishing to remove the tarnish caused by the heating.

Other studies suggested ultrasonic cleaning for 10 minutes followed by electropolishing for 45 seconds [9], ultrasonic cleaning for five minutes followed by electropolishing for 10 seconds [7], and sandblasting followed by electropolishing for 20 seconds [3]. However, in the present study, ultrasonic cleaning of the entire group of brackets (10 brackets) for 10 minutes followed by electropolishing for two minutes was done as per the manufacturer's recommendations given for the device used.

In the third recycling method, flame heating followed by sandblasting and electropolishing (RC III), previous studies showed that 90 µm aluminum oxide particle air abrasion at 90 PSI for 15-30 seconds was an effective method of recycling, which gave adequate bond strength [8]. However, sandblasting alone without flaming required more time to remove the adhesive, which could abrade the vulnerable undercuts. The time required for sandblasting the flamed mesh was shorter than that of removing the unburnt adhesive [7].

Earlier studies on the sandblasting method have suggested a Buchman method where brackets were flame heated for 5-10 seconds followed by sandblasting [3,6]. In the present study, flame heating was done for three to five seconds just to ignite the adhesives and sandblasted for 10-20 seconds to remove the adhesives completely. In a few earlier studies, sandblasting with 50 µm aluminum oxide particle stream at 4.5 bar pressure was used [6,7]. But this method showed lesser roughness and reduced bond strength when compared to 90 µm aluminum oxide particles stream sandblasting [8]. In the present study, the bracket base was kept at a distance of approximately 10 mm at 900 angulation causing no damage to the bracket surface [8]. Lack of electropolishing may cause corrosion due to surface roughness. To overcome this, electropolishing of the entire group of brackets was done for two minutes.

In a previous in vitro study on bacterial adhesion, Papaioannou et al. [20] assessed the whole bracket for adhesion. However, in a typical clinical situation, the bracket base adheres to the tooth surface, which will not be exposed to bacterial adhesion. Therefore, in the present study, the bracket was kept on red utility wax to prevent bacterial adhesion on the bracket base, as suggested in a study by Chen et al. [26].

Several species of bacteria are seen in the oral plaque biofilm but *Streptococcus mutans *was the main causative factor in enamel demineralization. Hence, *Streptococcus mutans* colonization was taken into consideration in the present study. In previous studies, selective medium mitis-salivarius agar was used to evaluate the *Streptococcus mutans* in plaque and saliva [15,27]. However, in the present study, 5% sheep blood agar, which is a nonselective medium, was used because only pure isolated strains of *Streptococcus mutans* (MTCC number: 497) were used.

In a few studies, metal brackets showed lower adhesion than ceramic and plastic brackets [16-18], but in contrast, other studies showed no difference in adhesion in various types of brackets [18-20]. The study results showed that the Group II brackets significantly showed more adhesion, Group I showed the least adhesion, and Group III brackets showed intermediate adhesion.

None of the recycled brackets in any of the groups showed significant results. The reason for the difference in the bacterial adhesion among the different groups could not be explained by the present study because factors like surface free energy characteristics [13,14], surface roughness [13,14], bracket material composition [13,14,28], presence of saliva to simulate oral environment [17,20], bracket design, size, and surface area of the different brackets were not assessed.

There was no statistically significant difference in *Streptococcus mutans* adhesion between new and recycled brackets in all three groups. This may be due to the fact that the assessment of CFUs gives only brief overall values and not the exact bacterial count. Therefore, using new advanced techniques like polymerase chain reaction (PCR) and further in vivo investigations could be considered.

## Conclusions

Since there is more *Streptococcus mutans* adhesion seen in all the recycled brackets in comparison to the new brackets, it is indicative of a change in the surface texture of recycled brackets. The recycling methods where electropolishing was incorporated showed lesser *Streptococcus mutans* adhesion. Statistical insignificance may be overcome by increasing the sample size. The molecular biological level study must be done in future studies. The statistical insignificance may be related to the incubation period. Hence, further in vitro and in vivo studies should be done.
